# New Drugs, Appliances, and Things Medical

**Published:** 1892-02-13

**Authors:** 


### NEW DRUGS, APPLIANCES, AND THINGS MEDICAL.

### THE INVIGORATOR CORSET.

The Invigorator corset, as supplied by Madame Reast, of Hastings, will prove of undoubted benefit to ladies, and above all growing girls suffering from weakness of the spine and who are inclined to stoop. The support afforded by them is most agreeable, no sense of constr int is experienced, and a feeling of added strength and res is produced. Mothers cannot do better than procure them or their daughters, and to nurses they will prove of great value, especial care being taken by means of elastic and padded straps that no rubbing of the delicate parts of the arm shall be the result of their use.

